# Treatment of Serous Effusions

**Published:** 1904-06

**Authors:** 


### ABSTRACTS.

### Treatment of Serous Effusions.

James Barr, in a clinical lecture delivered at the Liverpool Royal Infirmary, (*British Medical Journal*, March 19, 1904,) describes what is evidently a new method of treating serous effusions. The idea occurred to him to inject 1 fluidrachm of adrenalin chloride solution into the pleural sac, in a case of abdominal cancer extending to the pleura, after the aspiration of a large quantity of bloody serum, the object of the injection being to lessen the secretion. There was no further secretion, consequently no further tapping and the patient spent the remainder of her life in perfect comfort so far as her chest was concerned. This treatment was extended to cases of ascites due to hepatic cirrhosis in which marked results were not expected. However, the rapidity of secretion was diminished and no ill effects were noted, the quantity of adrenalin solution used varying from 2 to 3 fluidrachms.

In a case of pericarditis with effusion, in a lad, 19 fluidounces of serum were withdrawn from the pericardium, but a reaccumulation rapidly followed. The patient's condition becoming critical the paracentesis was repeated, 20 ounces of fluid being withdrawn with immediate improvement in the quality of the pulse. Forty minims of solution adrenalin chloride, 1-1000, were injected into the pericardium. The pulse at the wrist disappeared, the boy became of an ashy leaden hue and had an anxious expression. Immediately nitroglycerine and atropine were administered and the boy quickly rallied. No further tapping was required. The same patient had a subsequent attack of left pleurisy with effusion. Ten fluidounces of serum was withdrawn from the chest and 1 fluidrachm of adrenalin chloride solution was injected. There was no reaccumulation.

In a case of tuberculous peritonitis and ascites 200 fluidounces of serum was drawn and 2 fluidrachms of solution adrenalin chloride introduced into the peritoneal cavity, with 4 pints of aseptic air (to prevent adhesions). Thirteen days later 237 fluidounces of serum were withdrawn and 2 fluidrachms of adrenalin chloride solution and 2 pints of air were injected. Upon a third occasion, eleven days later, 196 fluidounces of serum was obtained by tapping, and 3 fluidrachms of adrenalin chloride solution and 4 pints of sterile air were injected. No reaccumulation of fluid occurred.

A female child of seven years was the next patient. One pint of fluid was withdrawn from her pleural cavity and 1 fluidrachm of adrenalin chloride solution and ½ pint of sterile air were injected. Though it was highly probable that the pleurisy was tuberculous there was no reaccumulation of fluid and the patient recovered.

